# Okanin Inhibits Cell Growth and Induces Apoptosis and Pyroptosis in Oral Cancer

**DOI:** 10.3390/cancers16183195

**Published:** 2024-09-19

**Authors:** Wei-Tso Chia, Kuei-Yuan Chen, Cheng-Yu Yang, Cheng-Chih Hsieh, Chang-Huei Tsao, Chih-Kung Lin, Bo Peng, Sien-Lin Ho, Yi-Ling Chen, Szu-Chien Chang, Yuan-Wu Chen

**Affiliations:** 1 Department of Orthopedics, National Taiwan University Hospital Hsin-Chu Branch, Hsinchu 302, Taiwan; 4926602@yahoo.com.tw; 2 Department of Nursing, Yuan Pie University of Medical Technology, Hsinchu 302, Taiwan; 3 Tri-Service General Hospital, Taipei 114, Taiwan; 4 School of Dentistry, National Defense Medical Center, Taipei 114, Taiwan; 0810ken@yahoo.com.tw (K.-Y.C.); hslcuyang@gmail.com (C.-Y.Y.); zx465077@gmail.com (B.P.); injection63@gmail.com (S.-L.H.); 5 Department of Oral and Maxillofacial Surgery, Tri-Service General Hospital, Taipei 114, Taiwan; 6 Department of Pharmacy, Kaohsiung Veterans General Hospital, Kaohsiung 813, Taiwan; p75phr60@gmail.com; 7 School of Pharmacy and Institute of Pharmacy, National Defense Medical Center, Taipei 114, Taiwan; 8 Department of Microbiology and Immunology, National Defense Medical Center, Taipei 114, Taiwan; changhuei@mail.ndmctsgh.edu.tw; 9 Department of Medical Research, Tri-Service General Hospital, Taipei 114, Taiwan; 10 Division of Anatomic Pathology, Taipei Tzu Chi Hospital, New Taipei City 231, Taiwan; doc31795@yahoo.com.tw; 11 Department of Dentistry, Kaohsiung Armed Forces General Hospital, Kaohsiung 813, Taiwan; m97510051225@gmail.com

**Keywords:** oral cancer, okanin, apoptosis, pyroptosis

## Abstract

**Simple Summary:**

Oral cancer is a challenging disease to treat, and new therapies are needed to improve patient outcomes. Okanin, a natural compound derived from *Bidens pilosa* L., has been known for its anti-inflammatory properties, but its effects on cancer, particularly oral cancer, are less understood. In this study, we investigated the anticancer potential of okanin in human oral cancer cells. Our results showed that okanin effectively reduced the growth of oral cancer cells by inducing cell death through mechanisms involving both apoptosis and pyroptosis. Additionally, okanin inhibited tumor growth in a mouse model of oral cancer. These findings suggest that okanin may be a promising natural compound for developing new treatments for oral cancer.

**Abstract:**

Background: Okanin, a flavonoid compound derived from *Bidens pilosa* L., has garnered attention for its anti-inflammatory properties. Although *Bidens pilosa* is commonly used in healthcare products and functional foods, the anticancer potential of okanin, particularly in oral cancer, remains underexplored. This study aims to investigate the effects of okanin on oral cancer cell lines and its potential as a therapeutic agent. Methods: The study involved assessing the cytotoxic effects of okanin on oral cancer cell lines SAS, SCC25, HSC3, and OEC-M1. The IC50 values were determined using methylene blue assays, and the clonogenic capacity was evaluated through colony formation assays. Flow cytometry was used to analyze cell cycle progression and apoptosis. Caspase-3/7 activity assays and annexin V/7-AAD staining confirmed the induction of apoptosis and pyroptosis. In vivo efficacy was assessed using a SAS xenograft model, and immunohistochemical analysis of xenograft tissue was performed to examine pyroptosis-related markers. Results: Okanin exhibited potent cytotoxic effects with IC50 values of 12.0 ± 0.8, 58.9 ± 18.7, 18.1 ± 5.3, and 43.2 ± 6.2 μM in SAS, SCC25, HSC3, and OEC-M1 cells, respectively. It caused dose- and time-dependent reductions in cell viability and significantly impaired clonogenic capacity. Flow cytometry revealed G2/M cell cycle arrest and increased sub-G1 population, indicating cell cycle disruption and death. Okanin induced both apoptosis and pyroptosis, as confirmed by caspase-3/7 activity and annexin V/7-AAD staining. In vivo, okanin reduced tumor growth and involved pyroptosis-related markers such as CASP1, GSDMC, GSDMD, and GSDME. Conclusions: Okanin demonstrates significant anticancer potential, particularly in oral cancer, by inducing both apoptosis and pyroptosis. Its efficacy in reducing tumor growth in vivo further supports its potential as a novel therapeutic option. Further mechanistic studies are needed to elucidate the pathways involved in okanin-mediated cell death and to explore its clinical applications.

## 1. Introduction

Head and neck squamous cell carcinomas (HNSCCs) originating from the epithelial lining of the oral cavity, hypopharynx, oropharynx, and larynx affect over 50,000 individuals annually in the United States [1] and more than 600,000 people worldwide [2]. Oral squamous cell carcinoma (OSCC), a malignant tumor of the oral cavity, presents significant challenges in diagnosis and treatment due to the associated cosmetic and functional morbidity. Recurrent or metastatic OSCC typically has a poor prognosis and is often incurable. Cisplatin, an alkylating agent that induces DNA damage and leads to cell cycle arrest and apoptosis, is considered the first-line treatment for advanced OSCC. However, its efficacy is limited by factors such as inherent and acquired resistance, as well as severe side effects including nephrotoxicity, peripheral neuropathy, and myelosuppression [3,4]. These limitations highlight the urgent need for novel therapeutic strategies for OSCC.

*Bidens pilosa* L., a plant traditionally used in various regions around the world for treating immune-related disorders, has gained attention for its medicinal properties [5]. The bioactive flavonoid monomer okanin, found in *Bidens pilosa*, has been reported to exhibit various pharmacological effects, including antithrombotic [6], antioxidant [7,8,9], and anti-inflammatory activities [10,11]. Okanin’s structure, characterized by an α-β unsaturated carbonyl group, plays a crucial role in its biological activity. For instance, studies have shown that this structural feature allows okanin to induce heme oxygenase-1 (HO-1) expression via the activation of nuclear factor-erythroid 2-related factor 2 (Nrf2) in macrophages, thereby inhibiting nitric oxide production and inducible nitric oxide synthase (iNOS) expression [12]. The Nrf2-ARE signaling pathway, which regulates the expression of cytoprotective enzymes, is of particular interest in cancer chemoprevention as it contributes to the detoxification and elimination of reactive intermediates formed from carcinogens [13].

Despite the known anti-inflammatory and antioxidant properties of okanin, its antitumor effects remain underexplored. In this study, we investigated the potential of okanin to inhibit the growth of oral cancer cells in four human oral cancer cell lines: SAS, SCC25, HSC3, and OEC-M1. We examined the cytotoxic effects of okanin and its influence on the expression of various apoptotic pathway-related factors in the SAS oral cancer cell line.

## 2. Materials and Methods

### 2.1. Reagents

The following chemicals and reagents were procured from Sigma-Aldrich (St. Louis, MO, USA): methylene blue (32723), Dimethyl sulfoxide (DMSO; C6164), pan-caspase inhibitor zVAD-FMK (627610), Kolliphor^®^ EL (C5315), and cisplatin (479306). Okanin (FO66168) was obtained from Biosynth Carbosynth (Compton, UK). The okanin stock concentration was 100 mg/mL dissolved in DMSO. The cisplatin stock concentration was 3 mM dissolved in sterile saline (0.9% NaCl). The administration solution formula is DMSO/Kolliphor/PBS (1:2:7).

### 2.2. Cell Lines and Culture

The SAS (JCRB0260; JCRB), SCC25 (CRL-1628; ATCC), and HSC3 cell lines are human oral squamous cell carcinoma cell lines. The SAS cell line was generously provided by Dr. Lo from the Institute of Oral Biology, Department of Dentistry, National Yang-Ming University, Taipei, Taiwan. The SCC25 cell line was obtained from the American Type Culture Collection (ATCC, Manassas, VA, USA). The HSC3 cell line was kindly provided by Dr. Yeh from the Department of Hematology and Oncology, Cancer Center, Taipei Medical University. The OEC-M1 cell line was generously provided by Dr. Jenn-Han Chen from the School of Dentistry, National Defense Medical Center, Taipei, Taiwan. All cell lines were cultured in RPMI 1640 (Capricorn Scientific, Ebsdorfergrund, Germany) supplemented with 10% fetal bovine serum (Biological Industries, Beit-Haemek, Israel), 1% penicillin/streptomycin, and 2 mmol/L L-glutamine in a humidified incubator with 5% CO_2_ at 37 °C.

### 2.3. Growth Inhibition Assay

Cells were cultured at a density of 10,000 cells per well in a 24-well plate and exposed to various concentrations of okanin for 48 h. The effect of okanin on cell growth was evaluated using the methylene blue dye assay (5 g per L of 50% (*v*/*v*) ethanol), as previously described [14]. The IC_50_ value was calculated graphically using GraphPad software (version 8.0) by comparing treated cells to the control group. Negative control: cells treated with vehicle (DMSO) only. Positive control: cells treated with cisplatin at its respective IC_50_ values (approximately 2 µM for SAS cells and 10 µM for SCC25 cells).

### 2.4. Plate Colony Formation Assay

SAS and SCC25 oral cancer cells were treated with okanin overnight before being seeded into a 6-well plate at a density of 1000 cells per well and maintained for 7 days. Negative controls (cells without okanin treatment) and positive controls (cells treated with cisplatin at their respective IC_50_ values, approximately 2 µM for SAS and 10 µM for SCC25) were included in the assay for comparison. The IC_50_ values for okanin were approximately 12.5 µM for SAS and 50 µM for SCC25. These concentrations were chosen based on preliminary growth inhibition assays to provide a range that includes lower and higher concentrations than the IC_50_, allowing for a comprehensive evaluation of okanin’s inhibitory effects on colony formation. This continuous exposure ensured that we could assess the compound’s effect on the ability of single cells to grow into colonies. The staining protocol was adapted from a previous study [15]. Colonies were stained with 0.5% methylene blue dye. The medium was pipetted off, and methylene blue solution was added directly to each well. The plate was gently swirled on the bench to ensure even mixing and left at room temperature for 30 min. After staining, the dye was pipetted off, and the wells were carefully rinsed with water. The plate was then inverted and left to dry on the bench. Images were captured, and the colonies were automatically counted using VisionWorks software (version 8.20, Analytik Jena, Upland, CA, USA).

### 2.5. Cell Cycle Analysis

SAS oral cancer cells were seeded at a density of 5 × 10^5^ cells per well in a six-well plate and cultured overnight under optimal conditions (37 °C, 5% CO_2_, 95% humidity). The cells were then treated with various concentrations of okanin, ranging from one-fourth to twice the IC_50_ value determined from the growth inhibition assay. This range was selected to observe the effects of sub-lethal, near-lethal, and supra-lethal doses on the cell cycle, thereby providing a comprehensive understanding of how okanin influences cell cycle dynamics at varying levels of efficacy. After 48 h, the cells were collected, fixed in 1 mL of 70% ethanol, and stored overnight at −20 °C. The next day, the fixed cells were washed with cold PBS and centrifuged at 2000 rpm for 10 min, and the supernatant was removed. The cell pellet was resuspended in PBS and stained with PI/RNase Staining Buffer (550825, BD, Becton Dickinson, Franklin Lakes, NJ, USA). The cells were incubated for 15 min at room temperature in the dark. Cell cycle phase distribution was analyzed using a FACSCalibur flow cytometer (BD biosciences, San Jose, CA, USA) with Kaluza Analysis 2.0 Software (Beckman Coulter, Brea, CA, USA).

### 2.6. Caspase-3 and -7 Activity Assay

Caspase-3 and -7 activities were assessed using FAM-FLICA^®^ Caspase Assay kits (ImmunoChemistry Technologies, Davis, CA, USA) according to the manufacturer’s instructions. SAS oral cancer cells were incubated with okanin at concentrations of 10 μM, 12.5 μM, and 20 μM for 48 h. Following incubation, the cells were collected, washed twice with cold PBS, and resuspended in Apoptosis Wash Buffer to a final concentration of 5 × 10^5^ cells/mL. Then, 290 μL of the cell suspension was transferred into tubes, and 10 μL of FLICA solution (diluted 1:5 *v*/*v* with PBS immediately before use) was added to the cells. The mixture was pipetted to mix and incubated in the dark at 37 °C for 1 h. After incubation, the cells were washed twice with 2 mL of Apoptosis Wash Buffer, centrifuged, and resuspended in 300 μL of the buffer. The samples were then immediately analyzed using a FACSCalibur flow cytometer (BD biosciences, San Jose, CA, USA) with Kaluza Analysis 2.0 Software (Beckman Coulter, Brea, CA, USA).

### 2.7. Apoptosis Assay

Cells were seeded at a density of 5 × 10^5^ cells/mL and treated with 12.5 µM okanin for 48 h. After treatment, the cells were collected by centrifugation and separated from the supernatant. The cell pellets were then washed with PBS and stained using the FITC Annexin V Apoptosis Detection Kit with 7-AAD (35-6410, Cytek^®^ Biosciences, Fremont, CA, USA) following the manufacturer’s instructions. Subsequently, the cells were analyzed using a FACSCalibur flow cytometer (BD biosciences, San Jose, CA, USA) with Kaluza Analysis 2.0 Software (Beckman Coulter, USA). Cells positive for annexin V and negative for 7-AAD were classified as apoptotic, while cells positive for both annexin V and 7-AAD were classified as pyroptotic [16]. Negative control: cells treated with vehicle (DMSO) only. Positive control: cells treated with cisplatin at its respective IC50 values (approximately 2 µM for SAS cells and 10 µM for SCC25 cells).

### 2.8. Quantitative Real-Time PCR

Total RNA was extracted from oral cancer cells using TRIzol Reagent (Invitrogen, Thermo Fisher Scientific, Waltham, MA, USA) following the manufacturer’s instructions. First-strand complementary DNA (cDNA) synthesis was performed using the Maxima H Minus First Strand cDNA Synthesis Kit (Thermo Scientific, Rockford, IL, USA). Quantitative real-time PCR (Q-PCR) was carried out using primers designed via Primer-BLAST (NCBI) for the following genes: CASP1 (CASP1-F: 5′-GCTGAGGTTGACATCACAGGCA-3′ and CASP1-R: 5′-TGCTGTCAGAGGTCTTGTGCTC-3′); CASP3 (CASP3-F: 5′-GGAAGCGAATCAATGGACTCTGG-3′ and CASP3-R: 5′-GCATCGACATCTGTACCAGACC-3′); CASP11 (CASP11-F: 5′-CGAGGCAGAAATTCTTCAGGTCC-3′ and CASP11-R: 5′-GCTGAGAACCATCAACTTGCTGG-3′); GSDMB (GSDMB-F: 5′-CCAGCAGTATCTGGCTACCCTT-3′ and GSDMB-R: 5′-CCTCCTTTACCGTCTCCAGAGT-3′); GSDMC (GSDMC-F: 5′-CCCATCACCAAACCTGGAAGAC-3′ and GSDMC-R: 5′-TCAACAGCCTCTGTCACCACGT-3′); GSDMD (GSDMD-F: 5′-ATGAGGTGCCTCCACAACTTCC-3′ and GSDMD-R: 5′-CCAGTTCCTTGGAGATGGTCTC-3′); GSDME (GSDME-F: 5′-GATCTCTGAGCACATGCAGGTC-3′ and GSDME-R: 5′-GTTGGAGTCCTTGGTGACATTCC-3′); IL1β (IL1β-F: 5′-CCACAGACCTTCCAGGAGAATG-3′ and IL1β-R: 5′-GTGCAGTTCAGTGATCGTACAGG-3′); IL18 (IL18-F: 5′-GATAGCCAGCCTAGAGGTATGG-3′ and IL18-R: 5′-CCTTGATGTTATCAGGAGGATTCA-3′); and GAPDH (GAPDH-F: 5′-GTCTCCTCTGACTTCAACAGCG-3′ and GAPDH-R: 5′-ACCACCCTGTTGCTGTAGCCAA-3′). Q-PCR amplifications were performed using the QuantStudio 5 Real-Time PCR System (Applied Biosystems, Waltham, MA, USA) in 20 μL reaction volumes containing 15 μL of SYBR Green PCR Master Mix (Thermo Scientific). The relative changes in mRNA expression were calculated using QuantStudio^TM^ Design and Analysis software (Version 1.5.2, Applied Biosystems, Thermo Scientific).

### 2.9. Western Blot Analysis

Cells were lysed in RIPA buffer containing 50 mM Tris (pH 7.8), 0.15 M NaCl, 5 mM EDTA, 0.5% Triton X-100, 0.5% NP-40, and 0.1% sodium deoxycholate, supplemented with a protease inhibitor mixture and a phosphatase inhibitor mixture (Calbiochem, Billerica, MA, USA). Protein concentration in the supernatants was determined using a BCA protein assay kit (Thermo Scientific). For each lane of 10% SDS-PAGE, 30 µg of cell lysate protein was loaded, separated, and transferred onto a polyvinylidene fluoride (PVDF) membrane (GE Healthcare, Hatfield, UK). The membranes were blocked with 5% non-fat dry milk in TBST and probed with primary antibodies: anti-CASP1 (Abcam, Cambridge, UK, ab62698), anti-GSDMC (GeneTex, Irvine, CA, USA, GTX33979), anti-GSDMD (Novus, Chesterfield, MO, USA, NBP2-33422), anti-GSDME (GeneTex, GTX64590), anti-cleaved N-terminal GSDMD (Abcam, ab215203), and anti-CASP3, CASP7, CASP9, and PARP (Cell Signaling Technology, Danvers, MA, USA, apoptosis antibody sampler kit #9915). After incubation with the primary antibodies, membranes were washed and incubated with HRP-conjugated secondary antibodies. Protein bands were visualized using an ECL detection system (GE Healthcare, UK) and captured using VisionWorks software (Analytik Jena, USA).

### 2.10. ELISAs (Enzyme-Linked Immunosorbent Assays)

Cells were treated with okanin for the indicated time points. Culture media were collected and centrifuged at 300× *g* for 10 min to remove cell debris and suspended cells. The supernatants were then transferred to fresh tubes and stored at −80 °C until analysis. IL-1β and IL-18 levels in the culture media were measured using the Human IL-1β ELISA Kit (900-T95, PeproTech, part of Thermo Fisher Scientific) and Human IL-18 ELISA Kit (KA0561, Abnova, Taipei, Taiwan), respectively, according to the manufacturers’ protocols. Briefly, samples and standards were added to 96-well plates pre-coated with capture antibodies, followed by incubation with detection antibodies and substrate solution. Absorbance was measured at 450 nm using a microplate reader, and cytokine concentrations were determined by comparison with standard curves.

### 2.11. Xenograft Tumor Model

The in vivo experiments were conducted with strict adherence to ethical guidelines, as approved by the National Defense Medical Center Institutional Animal Care and Use Committee, Taipei, Taiwan (IACUC 21-128). Six-week-old NOD.CB17 Prkdcscid/J mice (National Laboratory Animal Center, Taiwan) were housed in pathogen-free conditions within a microisolator. For tumor development, each mouse received a subcutaneous injection of 2 × 10^6^ SAS cells. After three days, the mice were randomly assigned into one of three groups, each consisting of five mice. One group was administered okanin (20 mg/kg) daily via intraperitoneal (i.p.) injection, another group received cisplatin (5 mg/kg) daily via i.p. injection, and the third group was given PBS as a vehicle control. Okanin and cisplatin were prepared in PBS, with okanin dissolved freshly before each use, while cisplatin was prepared at a concentration of 1 mg/mL and used immediately. All equipment and materials for drug administration were sterilized by autoclaving, and the solutions were filtered through a 0.22 µm sterile filter to maintain sterility. Mice were monitored daily for signs of distress or adverse effects, with body weight and tumor volume recorded twice a week. Tumor size was measured using Vernier calipers, and tumor volume was calculated using the following formula: volume = (length × width²)/2. The study lasted for a total of 4 weeks, including a 1-week period for tumor establishment followed by 3 weeks of treatment. At the end of the treatment period, mice were euthanized, and tumors were excised, weighed, and analyzed.

### 2.12. Histology and Immunohistochemistry

Mice were sacrificed using CO_2_, and their tissues were fixed through perfusion by using 4% paraformaldehyde in 0.1 M phosphate buffer. Next, 5 µm thick serial histological sections were taken on slides, deparaffinized in xylene, and rehydrated. After blocking endogenous peroxidase by using 3% hydrogen peroxide, the slides were incubated with the anti-CASP1 (1:100; Abcam, ab62698), anti-GSDMC (1:100; GeneTex, GTX33979), anti-GSDMD (1:100; Novus, NBP2-33422), and anti-GSDME (1:100; GeneTex, GTX64590) antibodies overnight at 4 °C. Target protein expression was detected using an antimouse and antirabbit peroxidase complex, and peroxidase activity was observed using 3-amino-9-ethyl-carbazole. The slides were counterstained with hematoxylin (Sigma-Aldrich) and mounted using a mounting solution.

### 2.13. Statistical Analysis

All statistical analyses were performed using the built-in functions of GraphPad software (version 8.0). Data are presented as the mean ± standard deviation (SD). For comparisons between two groups, statistical significance was determined using the Student’s *t*-test. A *p* value of <0.05 was considered statistically significant.

## 3. Results

### 3.1. Okanin Inhibits the Viability and Colony Formation of OSCC Cells

**Figure 1:**
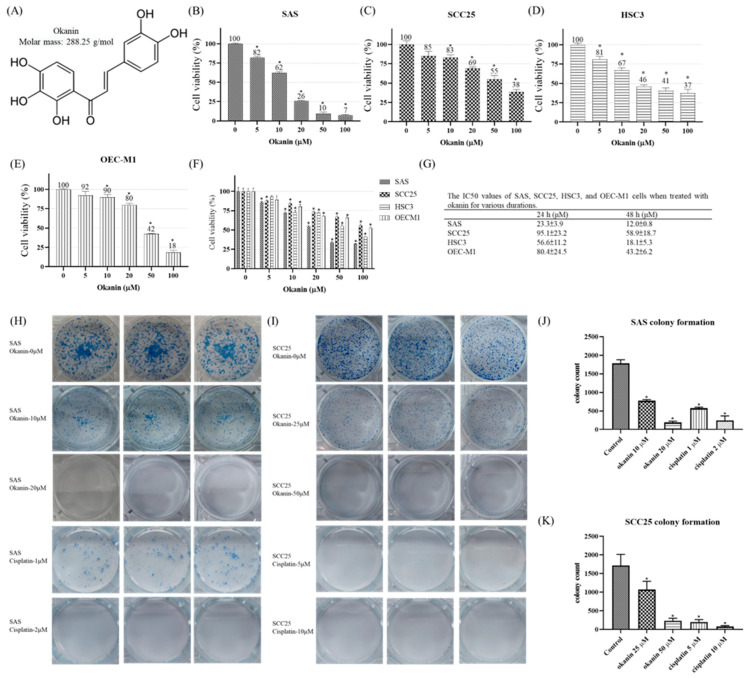
Cytotoxic effects of okanin on SAS, SCC25, HSC3, and OEC-M1 cells. (**A**) The chemical structure of okanin. (**B**) SAS, (**C**) SCC25, (**D**) HSC3, and (**E**) OEC-M1 cells were treated with varying concentrations of okanin for 48 h. (**F**) The four oral cancer cell lines were treated with okanin for 24 h, and (**G**) the IC50 values were determined. (**H**) Representative images from the plate colony formation assay for SAS cells, and (**I**) SCC25 cells. (**J**) and (**K**) Quantitative analyses of colony formation in SAS and SCC25 cells, respectively. Cisplatin was used as a positive control. Asterisks (*) denote statistically significant differences compared to the control group (* *p* < 0.05). Each experiment was performed in triplicate.

To assess the effects of okanin on OSCC cells, a cell viability assay was conducted. Treatment with varying concentrations of okanin resulted in a dose- and time-dependent reduction in cell viability across SAS, SCC25, HSC3, and OEC-M1 cell lines, as revealed by the methylene blue assay (Figure 1). A gradual decrease in cell viability was observed in oral cancer cells with increasing concentrations of okanin compared to non-treated cells (Figure 1B–F). Cisplatin was used as a positive control group (Supplementary Figure S1). The IC_50_ values for the four oral cancer cell lines are presented in Figure 1G, indicating that SAS cells were particularly sensitive to okanin. Additionally, the colony formation efficiency of okanin-treated OSCC cells was examined (Figure 1H–K). The results demonstrated a dose-dependent reduction in colony formation in SAS (Figure 1H,J) and SCC25 (Figure 1I,K) cells upon exposure to okanin. Higher concentrations of okanin (20 μM and 50 μM) exhibited more pronounced inhibitory effects on OSCC cells. Overall, these findings highlight okanin’s dose-dependent ability to diminish both cell viability and colony formation in OSCC cells.

### 3.2. G2/M Arrest and Sub-G1 Fraction Induction by Okanin in SAS Cells

**Figure 2:**
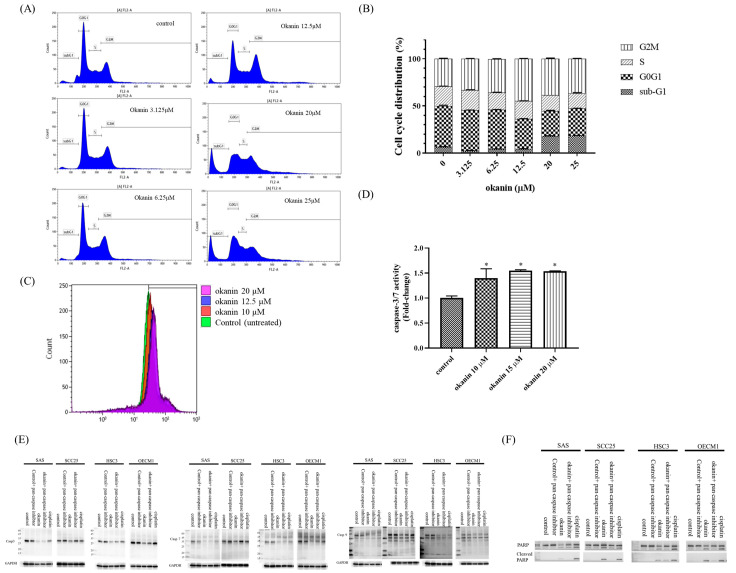
Okanin induces cell cycle arrest and apoptosis in oral cancer cells. (**A**) Flow cytometry analysis of SAS cells treated with various concentrations of okanin for 48 h, showing G2/M phase arrest. (**B**) Quantitative representation of cell distribution across different cell cycle phases. (**C**) Measurement of caspase-3/7 activity in SAS cells treated with okanin for 48 h using flow cytometry. (**D**) Fold change in caspase-3/7 activity after treatment with various concentrations of okanin for 48 h in SAS cells. (**E**) Protein expression levels of caspase-3, -7, -9, and PARP (**F**) were detected by Western blot analysis. Asterisks (*) denote statistically significant differences compared to the control group (* *p* < 0.05). Each experiment was performed in triplicate.

To investigate the mechanism behind the antiproliferative effect of okanin in SAS cells, the cell cycle distribution was analyzed. As shown in Figure 2, okanin increased the proportion of cells arrested in the G2/M phase in a concentration-dependent manner, while simultaneously decreasing the cell population in the G1 and S phases. Additionally, there was a significant increase in the number of cells in the apoptotic sub-G1 phase with increasing concentrations of okanin (Figure 2B).

To determine if okanin induces cell death in SAS oral cancer cells and to identify the mode of death, we performed a caspase-3/7 activity assay using flow cytometry (Figure 2C,D). The results showed that okanin increased caspase-3/7 activity. A Western blotting assay showed that okanin could inhibit the expression of pro-caspase-3, -7, and -9. In addition, okanin could induce cleaved PARP (Figure 2E).

### 3.3. Okanin Induces Pyroptosis in SAS Cells

**Figure 3:**
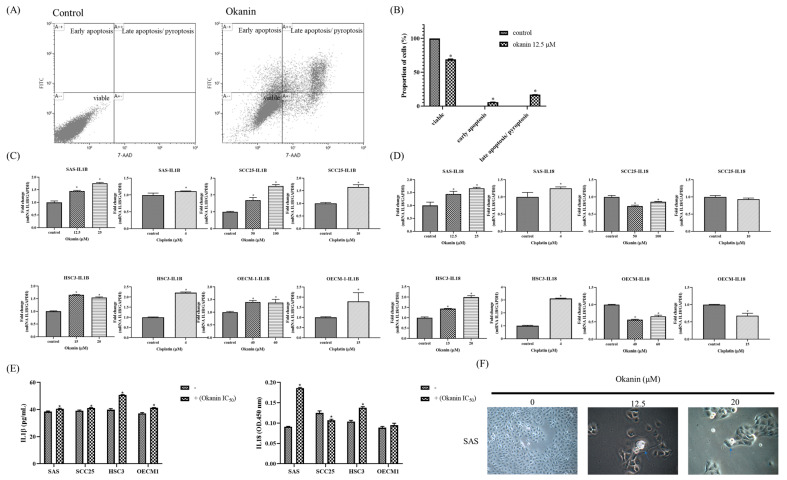
Okanin induces pyroptosis in oral cancer cells. (**A**) Apoptotic cell frequencies in SAS cells treated with or without okanin (12.5 μM) for 48 h, as determined by annexin V/7-AAD staining assays. (**B**) Quantification of annexin V/7-AAD staining in SAS cells following 48 h of okanin treatment. (**C**) IL-1β mRNA expression was assessed by qPCR. (**D**) IL-18 mRNA expression was assessed by qPCR. (**E**) The levels of IL-1β and IL-18 released into the culture medium were measured by ELISA. (**F**) Morphological changes induced by okanin treatment, with arrows indicating cell swelling and rupture. Asterisks (*) denote statistically significant differences compared to the control group (* *p* < 0.05). Each experiment was performed in triplicate.

**Figure 4:**
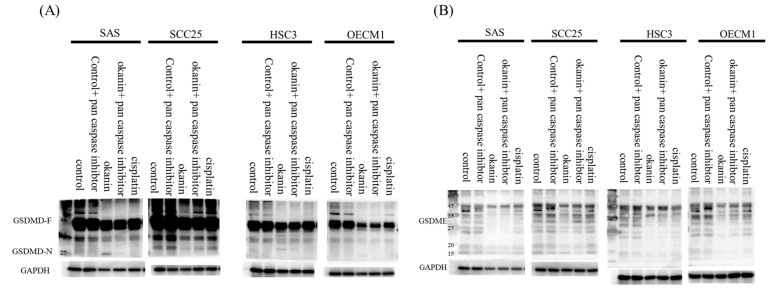
Okanin reduced the expression of GSDMD and GSDME in oral cancer cells. (**A**) Protein levels of full-length GSDMD and the GSDMD-N terminus, and (**B**) GSDME in cells treated with or without okanin (IC_50_), pan-caspase inhibitor (20μM), and cisplatin (IC_50_) for 48 h, were assessed by Western blot analysis.

To determine if okanin induces cell death in SAS oral cancer cells and to identify the mode of death, okanin-treated cells were stained with annexin V/7-AAD and analyzed by flow cytometry. Okanin significantly induced cell death in SAS cells, with the dead cells predominantly marked as annexin V+ 7-AAD+, indicating that the cell death involved both pyroptosis and apoptosis (Figure 3A,B). The IL-1β and IL-18 mRNA expression was assessed by qPCR (Figure 3C,D). The levels of IL-1β and IL-18 released into the culture medium were measured by ELISA (Figure 3E). Morphological alteration was observed in SAS and in OEC-M1 (Supplementary Figure S2). Furthermore, okanin reduced the expression of GSDMD and GSDME in oral cancer cells (Figure 4). Together, these data suggest that pyroptosis occurred after okanin treatment (Figure 3).

### 3.4. Effect of Okanin on Tumor Growth In Vivo

**Figure 5:**
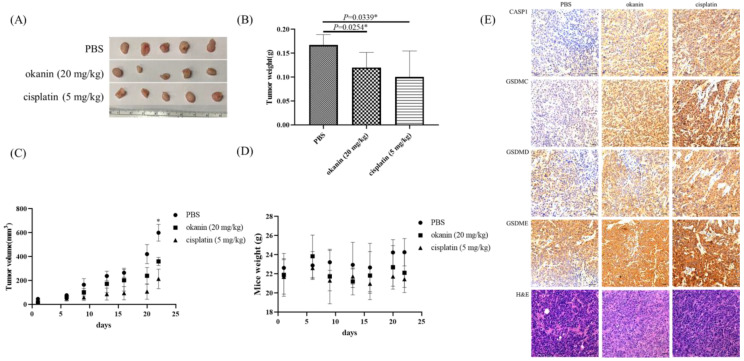
Okanin inhibits tumorigenicity of SAS cell in vivo. (**A**) Representative images of xenograft tumors at the endpoint of the experiment. (**B**) Average tumor weight across different treatment groups, with values presented as mean ± SD (*n* = 5). (**C**) Growth curve of xenograft tumors in mice treated with okanin (20 mg/kg, intraperitoneally) (*n* = 5/group). (**D**) Body weight of mice monitored during the experiment. Asterisks (*) denote statistically significant differences compared to the control group (* *p* < 0.05). (**E**) Hematoxylin and eosin (H&E) staining and immunohistochemical (IHC) staining of xenograft tissue sections. The H&E staining shows the general histological structure of the tumor sections, while IHC staining was performed for pyroptosis biomarkers, including CASP1, GSDMC, GSDMD, and GSDME.

Xenograft mouse models were utilized to evaluate the antitumor effect of okanin in vivo using SAS cells (Figure 5). The results demonstrated that okanin treatment significantly delayed the growth of SAS xenograft tumors, reducing tumor volume by approximately two-thirds compared to PBS-treated controls by day 21 (Figure 5A). No significant changes in body weight were observed among the groups (Figure 5B). Additionally, the tumor mass weight was reduced by about 30% in the okanin-treated group (Figure 5C,D). Immunohistochemical (IHC) staining of xenograft tissue sections for pyroptosis biomarkers, including CASP1, GSDMC, GSDMD, and GSDME (Figure 5E), was carried out. The expression biomarkers were higher in the okanin treatment group than in the PBS group. These findings indicate that okanin exerts an anticancer effect by inhibiting oral tumor growth in vivo. Okanin could induce pyroptosis in oral cancer xenografts.

## 4. Discussion

Cancer is characterized by several hallmarks, including sustaining proliferative signaling, evading growth suppressors, avoiding immune destruction, and promoting tumor-associated inflammation [17]. These hallmark capabilities have significantly influenced the development of novel cancer treatments [17]. Inflammation is closely associated with cancer, with many anticancer agents also being utilized in the treatment of inflammatory diseases [18]. The link between chronic inflammation and increased cancer risk underscores the potential of targeting inflammation as a strategy for cancer prevention and therapy [18].

Okanin, a bioactive compound from *Bidens pilosa* L., an edible herb traditionally used for various ailments, has demonstrated anti-inflammatory activity in microglia [10,19]. However, its anticancer potential remains largely unexplored. Our study aimed to investigate this potential, and we have demonstrated that okanin exhibits significant anticancer properties both in vitro and in vivo, although further studies are needed to elucidate the precise mechanisms involved.

The primary objective of cancer therapies is to induce tumor cell death. Non-invasive treatments focus on maximizing tumor cell death while minimizing toxicity to healthy tissues by targeting cancer-specific properties, such as elevated proliferation rates and activated pro-survival pathways. Many clinically used chemotherapeutics, such as paclitaxel [20], doxorubicin [21,22], cisplatin [21], BRAF inhibitors, MEK inhibitors [22], and sorafenib [23], have been reported to induce pyroptosis in tumor cells as part of their antitumor mechanisms.

Our results align with this body of research, suggesting that okanin may similarly induce pyroptosis in oral cancer cells. Immune checkpoint blockade therapy has shown promise in treating various malignancies, including oral cancer [24,25,26,27]. However, its efficacy is often limited due to the “immune cold” status of the tumor immune microenvironment [26]. Pyroptosis, a form of gasdermin-mediated programmed cell death, has been implicated in modifying the tumor immune microenvironment, potentially enhancing the effectiveness of immune checkpoint blockade therapies [28]. Therapies that promote pyroptosis and immune checkpoint blockade may have synergistic effects in cancer treatment [24,29]. Pyroptosis can be detected through several common methods [16]. By inducing pyroptosis, okanin may release tumor-associated antigens, damage-associated molecular patterns, and proinflammatory cytokines, leading to intratumoral inflammatory responses, increased tumor-specific cytotoxic T cell infiltration, and the conversion of “cold” tumors to “hot” tumors.

In our study, the induction of both apoptosis and pyroptosis by okanin in oral cancer cells was supported by flow cytometry analysis. The use of specific markers for pyroptosis, such as caspase-1 activation, gasdermin D cleavage, and the release of IL-1β, could further substantiate these findings. However, the underlying mechanisms and pathways through which okanin induces pyroptosis require further exploration.

## 5. Conclusions

In conclusion, this study is the first to unveil the previously unrecognized anticancer potential of the natural small molecule okanin. This discovery paves the way for new research directions, highlighting okanin as a promising therapeutic agent with significant potential in oral cancer treatment. The ability of okanin to induce apoptosis and pyroptosis in oral cancer cells marks a pivotal step forward in expanding its therapeutic applications. Given its multi-faceted biological activities, okanin emerges as a compelling candidate for further investigation and development in cancer therapy, offering novel strategies for targeting malignancies like oral squamous cell carcinoma.

## Data Availability

The data that support the findings of this study are available from the corresponding author upon reasonable request.

## Supplementary Materials

The following supporting information can be downloaded at https://www.mdpi.com/article/10.3390/cancers16183195/s1: Figure S1: Okanin and cisplatin inhibit the viability of OSCC Cells; Figure S2: Treatment of oral squamous cell carcinoma (OSCC) with okanin induces morphological characteristics typical of pyroptosis, such as cell swelling, membrane rupture, and the formation of pyroptotic bodies; Figure S3: Treating oral squamous cell carcinoma (OSCC) with okanin induces GSDM families’ gene expression changes; Figure S4. Treating oral squamous cell carcinoma (OSCC) with okanin induces GSDM families’ gene expression changes. (A) CASP1 mRNA expression fold change; Figure S5. Treating oral squamous cell carcinoma (OSCC) with okanin induces caspase1 (CASP1) expression changes in oral cancer cell lines; Figure S6. ROS assay for okanin treatment; Figure S7. Mitochondrial membrane potential assay for okanin treatment.
