# Supplementary material for: Okanin Inhibits Cell Growth and Induces Apoptosis and Pyroptosis in Oral Cancer
Source: Cancers (Basel). 2024 Sep 19;16(18):3195. doi: 10.3390/cancers16183195 (PMC11429813; doi:10.3390/cancers16183195)
Supplement: Supplementary file 1 [file cancers-16-03195-s001.zip › cancers-3180370-supplementary.pdf]

cancers

MDPI

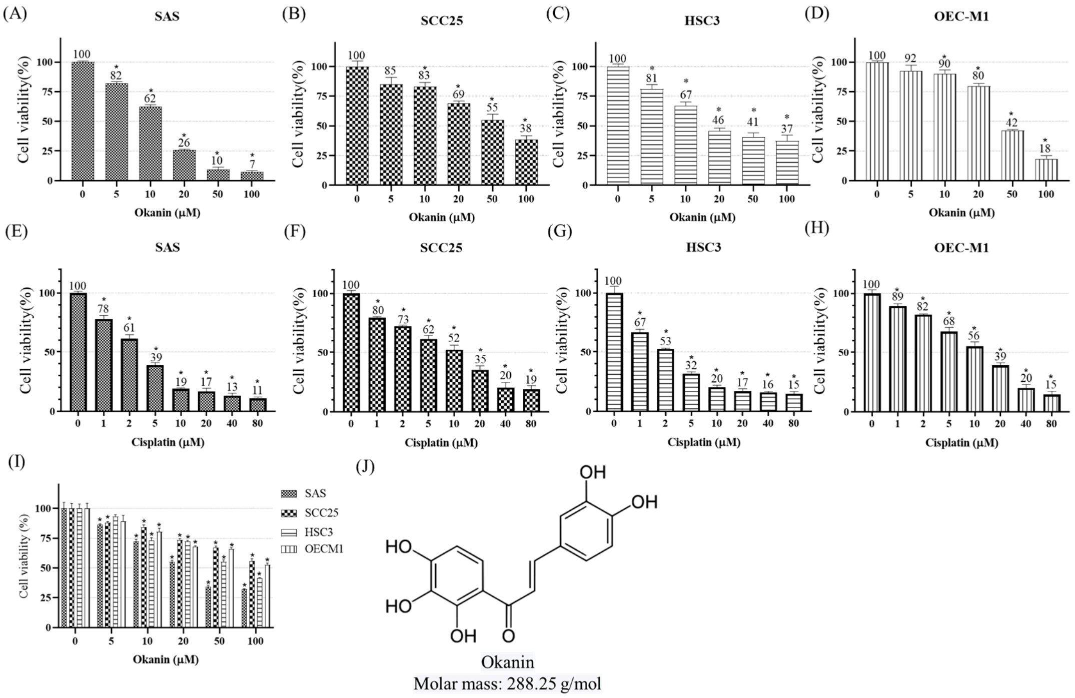

Figure S1. Okanin and cisplatin inhibit the viability of OSCC Cells.

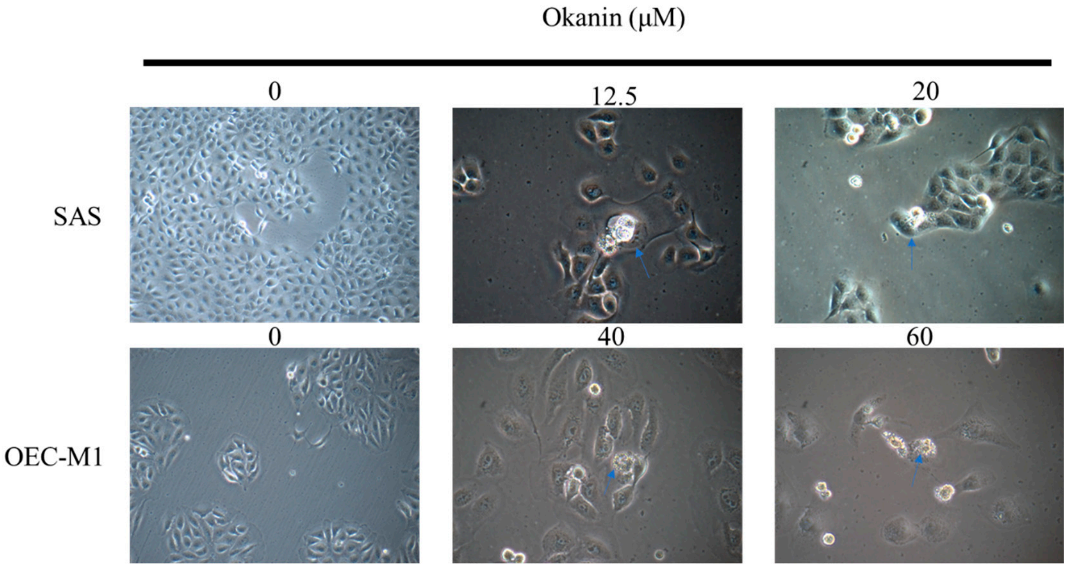

Figure S2. Treatment of oral squamous cell carcinoma (OSCC) with okanin induces morphological characteristics typical of pyroptosis, such as cell swelling, membrane rupture, and the formation of pyroptotic bodies.

Biochemical Markers: We evaluated the activation of pyroptosis-specific markers, including caspase-1 and gasdermin D cleavage. Western blot analysis, and qPCR analysis were performed. These observations have been added to the revised manuscript in the Supplementary Figures S2-S4.

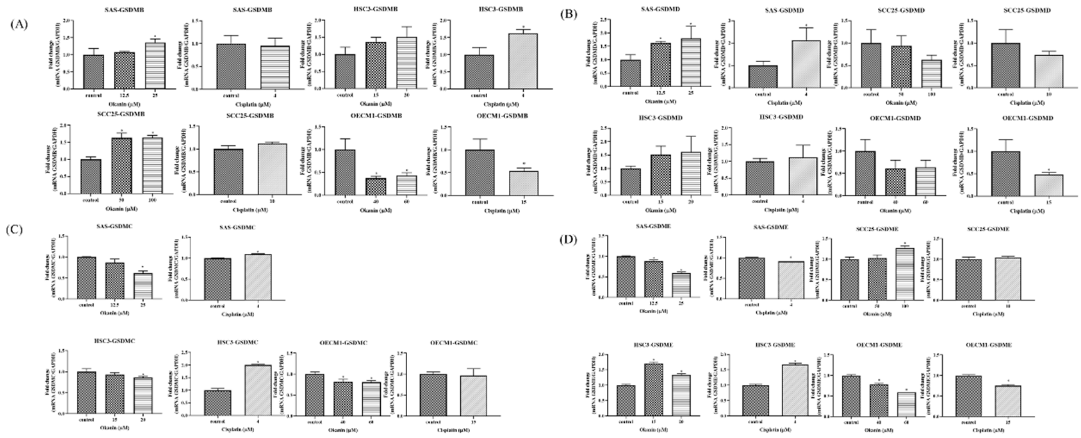

Figure S3. Treating oral squamous cell carcinoma (OSCC) with okanin induces GSDM families' gene expression changes. (A) GSDMB mRNA expression fold change. (B) GSDMD mRNA expression fold change (C) GSDMC mRNA expression fold change. (D) GSDME mRNA expression fold change in oral cancer cell lines.

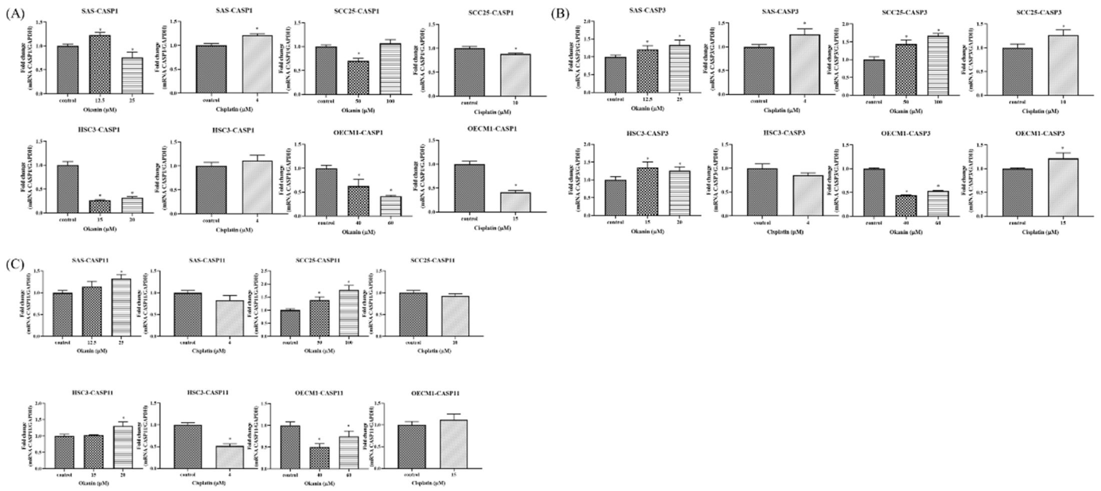

Figure S4. Treating oral squamous cell carcinoma (OSCC) with okanin induces GSDM families' gene expression changes. (A) CASP1 mRNA expression fold change. (B) CASP3 mRNA expression fold change (C) CASP11 mRNA expression fold change in oral cancer cell lines.

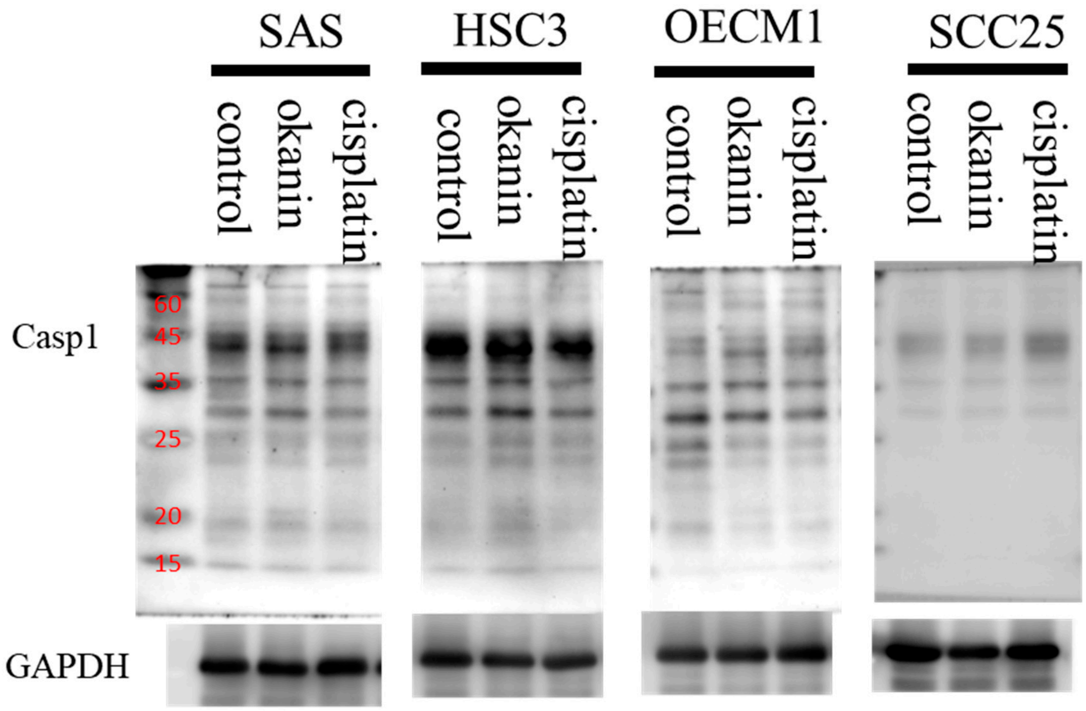

Figure S5. Treating oral squamous cell carcinoma (OSCC) with okanin induces caspase1 (CASP1) expression changes in oral cancer cell lines.

ROS-SAS

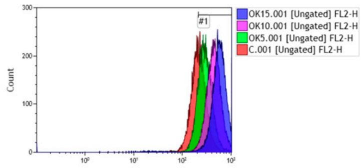

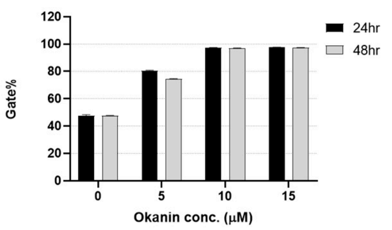

ROS-SCC25

ROS-48hr	SCC25	
Okanin(μM)	Gate%	X-Med
0	50.44±0.29	13.71±0.06
30	88.38±0.19	21.17±0.17
60	88.63±0.34	29.90±0.17
90	92.03±0.23	42.15±0.20

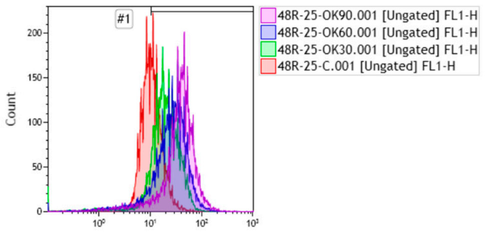

ROS-HSC3

ROS-24hr	HSC3	
Okanin(μM)	Gate%	X-Med
0	49.53±3.21	16.88±0.16
5	50.60±0.26	16.77±0.02
10	69.76±0.39	18.42±0.05
15	85.99±0.41	23.17±0.13
20	81.34±0.57	23.68±0.12

ROS-48hr	HSC3	
Okanin(μM)	Gate%	X-Med
0	51.72±0.18	15.10±0.08
5	63.89±2.49	16.55±0.14
10	68.81±2.39	16.46±0.30
15	85.49±0.14	22.10±0.15
20	85.34±0.31	24.89±0.20

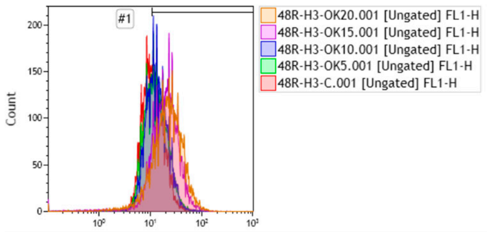

ROS-OECM1

ROS-48hr	OECM1	
Okanin(μM)	Gate%	X-Med
0	51.78±0.85	13.76±0.07
30	75.47±0.48	17.42±0.12
60	83.48±0.26	30.98±0.25
90	80.22±5.99	37.62±0.05

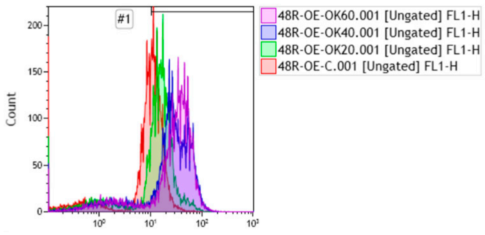

Figure S6. ROS assay for okanin treatment.

Mitochondrial membrane potential-SAS

MP-24hr	SAS	
Okanin(μM)	Gate%	X-Med
0	82.51±0.29	70.25±0.36
6.25	85.46±0.24	53.23±0.03
12.5	87.46±0.14	58.72±0.25
20	93.31±0.31	53.97±0.25
25	88.78±5.44	44.86±0.27

MP-48hr	SAS	
Okanin(μM)	Gate%	X-Med
0	91.03±0.38	32.52±0.30
6.25	91.03±0.04	29.83±0.10
12.5	87.87±0.61	48.10±0.25
20	94.03±0.14	105.07±0.41
25	93.47±0.21	88.22±0.56

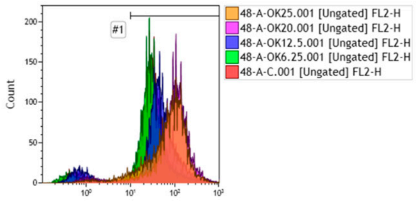

Mitochondrial membrane potential-SCC25

MP-48hr	SCC25	
Okanin(μM)	Gate%	X-Med
0	93.34±0.19	37.86±0.13
30	94.14±0.25	78.47±0.65
60	91.62±0.17	74.92±0.48
90	91.33±0.17	73.42±0.86

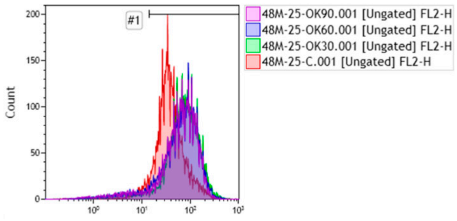

Mitochondrial membrane potential-HSC3

MP-24hr	HSC3	
Okanin(μM)	Gate%	X-Med
0	91.06±0.18	58.34±0.18
5	89.88±0.16	45.76±0.14
10	90.72±0.13	47.92±0.14
15	92.15±0.34	63.06±0.4+6
20	94.09±0.08	60.69±0.14

MP-48hr	HSC3	
Okanin(μM)	Gate%	X-Med
0	92.91±0.16	47.05±0.32
5	83.99±0.31	29.75±0.33
10	81.55±0.40	37.03±0.09
15	90.11±0.08	67.70±0.52
20	90.71±0.23	98.55±1.36

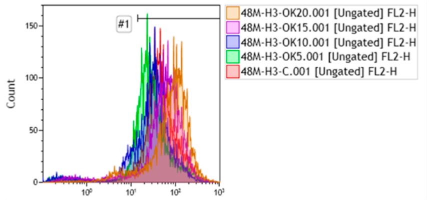

Mitochondrial membrane potential-OECM1

MP-24hr	**OECM1**	
Okanin(μM)	Gate%	X-Med
0	92.70±0.28	58.34±0.19
20	95.68±0.22	77.55±0.01
40	95.45±0.16	81.60±0.54
60	93.95±0.17	85.09±0.22

MP-48hr	**OECM1**	
Okanin(μM)	Gate%	X-Med
0	84.64±0.63	36.83±0.26
20	92.09±0.17	64.68±0.30
40	91.98±0.06	85.80±0.50
60	88.07±0.24	107.69±0.94

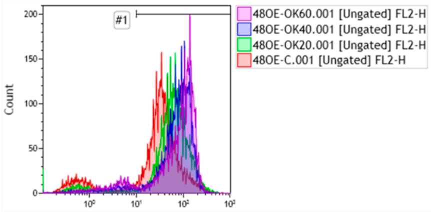

Figure S7. Mitochondrial membrane potential assay for okanin treatment.
